# Shape variation in the least killifish: ecological associations of phenotypic variation and the effects of a common garden

**DOI:** 10.1002/ece3.1780

**Published:** 2015-11-17

**Authors:** J. Alex Landy, Joseph Travis

**Affiliations:** ^1^ Department of Biological ScienceFlorida State UniversityTallahasseeFlorida

**Keywords:** Common garden, ecological trait associations, local adaptation, morphometrics, phenotypic variation, Poeciliidae

## Abstract

Studies of the adaptive significance of variation among conspecific populations often focus on a single ecological factor. However, habitats rarely differ in only a single ecological factor, creating a challenge for identifying the relative importance of the various ecological factors that might be maintaining local adaptation. Here we investigate the ecological factors associated with male body shape variation among nine populations of the poeciliid fish, *Heterandria formosa*, from three distinct habitats and combine those results with a laboratory study of three of those populations to assess the contributions of genetic and environmental influences to shape variation. Field‐collected animals varied principally in three ways: the orientation of the gonopodium, the intromittent organ; the degree of body depth and streamlining; and the shape of the tail musculature. Fish collected in the spring season were larger and had a more anteriorly positioned gonopodium than fish collected in autumn. Fish collected from lotic springs were larger and more streamlined than those collected from lentic ponds or tidal marshes. Some of the variation in male shape among populations within habitats was associated with population‐level variation in species richness, adult density, vegetative cover, predation risk, and female standard length. Population‐level differences among males in body size, position of the gonopodium, and shape of the tail musculature were maintained among males reared in a common environment. In contrast, population variation in the degree of streamlining was eliminated when males were reared in a common environment. These results illustrate the complicated construction of multivariate phenotypic variation and suggest that different agents of selection have acted on different components of shape.

## Introduction

Many different ecological factors have been found to promote differentiation among conspecific populations in behavioral, life history, and morphological traits (Reznick and Travis 1996; Travis and Reznick 1998; Schluter 2001; Rundle and Nosil 2005). A large proportion of this literature focuses on how specific phenotypic traits vary among habitats. Populations are often grouped into broad habitat types based on either a single biotic selective agent such as presence or absence of predators or a single abiotic contrast such as that between lentic and lotic bodies of water (Langerhans et al. 2004; Herczeg et al. 2010; Gaston and Lauer 2015). However, habitats rarely differ in only a single putative ecological agent of selection. For example, variation in predation pressure creates differences in a variety of other ecological factors such as population density of the prey, which could itself be an important agent of natural selection within the ecosystem (Bassar et al. 2012; Travis et al. 2014). The covariation among possible agents of selection often seen in contrasting habitats creates a challenge for identifying which among many ecological factors play a role in maintaining population differentiation and with what relative importance.

This is a difficult challenge to address without understanding the ecological genesis of selection pressures (Rundle and Nosil 2005). Detailed ecological study can reveal how the strength of individual agents of selection can vary temporally (Trexler et al. 1992; Reimchen and Nosil 2002) or be contingent on the action of other ecological factors (Trexler et al. 1994). Ecological studies can also reveal how multiple agents of selection can interact and create synergistic effects on the net selection gradient on a phenotype (Travis et al. 1985; Sih et al. 1998).

The body shape of an animal is an especially interesting trait in this context because it is subject to several different forces of selection, some of which may covary among locations and might either reinforce or conflict with one another in the phenotypic features they favor. This paradigm is particularly true for fish, whose shape in a fluid medium is influenced by many factors, including predation pressure, population density, habitat complexity, water chemistry, and flow rate (Walker and Bell 2000; Hendry et al. 2006; Gomes and Monteiro 2008; Collin and Fumagalli 2011; Ruehl et al. 2011; Young et al. 2011; Bartels et al. 2012; Lostrom et al. 2015). Fish populations occurring in high flow areas are often thinner and have more streamlined bodies when compared to conspecifics found in low flow areas (Franssen 2011; Fu et al. 2013; Gaston and Lauer 2015). Foraging habits and the position of available food resources, whether they are suspended in the water column, floating on the surface or located in the benthos, have been found to affect morphological development in fishes (Robinson and Wilson 1995; Svanbäck and Eklöv 2002). Morphological differences have also been observed among fish populations in response to the presence or absence of specific predators (Walker 1997; Langerhans et al. 2004). Even within a single group like the poeciliid fishes, several agents of natural selection, as well as sexual selection, have been identified as acting individually on body shape (Grether and Kolluru 2011).

The least killifish, *Heterandria formosa*, is an ideal species for exploring the influence of covarying ecological factors on among‐population variation. *Heterandria formosa* is a poeciliid fish found in a variety of habitats and has been the subject of considerable ecological and evolutionary research (Travis et al. 1987; Henrich 1988; Baer 1998a,b; Leips and Travis 1999; Baer and Travis 2000; Baer et al. 2000; Leips et al. 2000, 2009; Soucy and Travis 2003; Richardson et al. 2006; Schrader and Travis 2009; Schrader et al. 2011; MacRae and Travis 2014; Hale and Travis 2015). Populations of *H. formosa* encounter different flow regimes, different thermal regimes, experience different risks of predation from different predator assemblages, and forage in locations with different levels of habitat complexity, making them especially suitable for dissecting how different ecological agents might contribute to the net selective force on body shape variation.

In this study, we (1) quantify and assess population differentiation in regard to body shape among nine populations of *H. formosa*, (2) test for covariation between population‐level phenotypic variation and the various ecological variables that characterize each location, and (3) conduct a common garden experiment to assess the phenotypic response of F1 male *H. formosa* to shared laboratory conditions.

## Methods

### Study system and sampling

**Figure 1:**
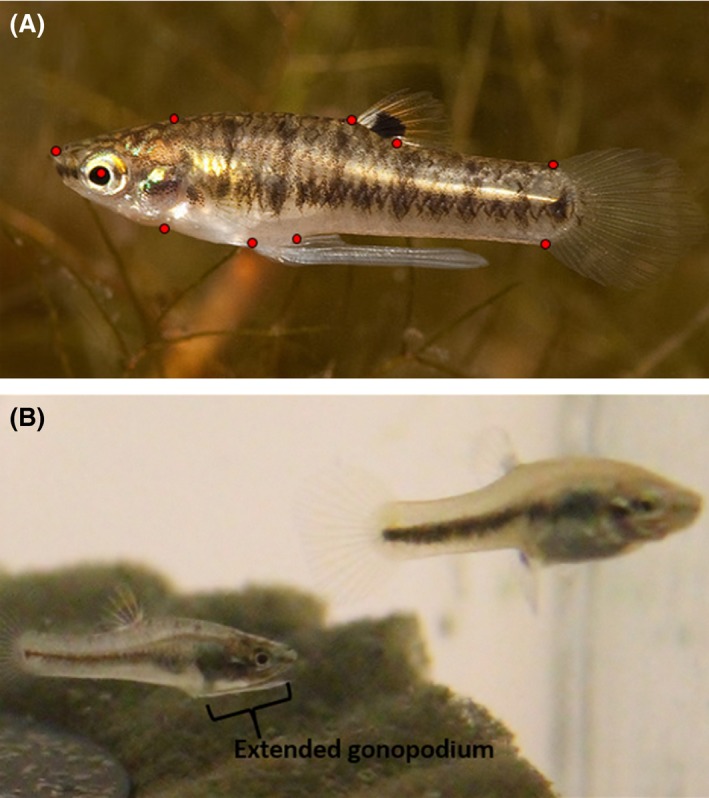
(A) Geometric morphometric landmark positions of *Heterandria formosa*: landmark one is located on the tip of the snout. Landmark two is on the supraoccipital crest. Landmarks 3 and 4 are on the anterior and posterior insertion points of the dorsal fin. Landmarks 5 and 6 are on the dorsal and ventral edge of the caudal fin. Landmarks 7 and 8 are on the posterior and anterior edges of the gonopodium. Landmark 9 is located on suspensorium. Landmark 10 is located on the center of the eye. (B) Male (bottom left) and female (top right) *H. formosa*. Male has positioned his gonopodium anteriorly in preparation for a mating attempt.

*Heterandria formosa* is native to the Coastal Plain of the southeastern United States. Males can grow up to 1.5 cm in length and females grow to 2.5 cm in length (Fig. 1A). They occupy the shallow littoral zone and feed primarily on periphyton (Aresco et al. 2015). Like all poeciliids, *H. formosa* males have an elongated anal fin, the gonopodium, which is used as the intromittent organ (Fig. 1B). Females are live‐bearing, exhibiting extreme degrees of both superfetation (carrying multiple developing broods simultaneously) and matrotrophy (providing substantial nourishment to embryos after fertilization) (Schrader and Travis 2009). Studies on the genetic population structure in multiple drainages suggest that local populations of *H. formosa* can easily evolve independently because they exchange migrants at an exceptionally low rate (Baer 1998b; Soucy and Travis 2003; Schrader et al. 2011).

**Figure 2:**
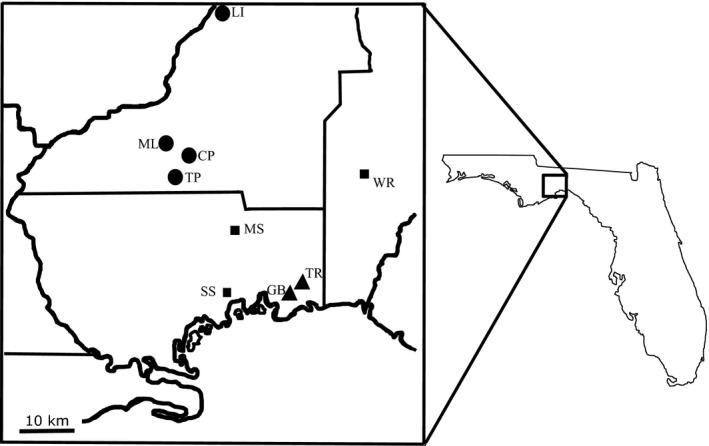
Map of *Heterandria formosa* populations in North Florida. Cessna Pond (CP), Lake Iamonia (LI), Moore Lake (ML), and Trout Pond (TP) are lentic pond habitats (circles). Gambo Bayou (GB) and Tram Road (TR) are freshwater intertidal marshes (triangles). McBride Slough (MS), Shepherd Spring (SS), and Wacissa River (WR) are lotic springs (squares).

We studied nine *H. formosa* populations from three distinct habitats in Jefferson, Leon, and Wakulla counties of North Florida (Table 1; Fig. 2). Three of these populations are lotic springs with hard basic water, four populations are lentic ponds with soft acidic water, and two populations are freshwater tidal marshes within the St. Mark's National Wildlife Refuge. There are substantial differences among these habitats in abiotic factors (Leips and Travis 1999; MacRae and Travis 2014). The atmospheric temperatures are similar at all nine populations throughout the year; annual and daily fluctuations in water temperature are greater in ponds, in which temperatures can exceed 30°C in summer and be as low as 5°C on some winter days. The water temperature in the springs is buffered from large fluctuations because of inflow into the springs from the Florida aquifer, in which the water flows at about 20°C throughout the year. Freshwater marshes are intermediate in most of these ecological parameters.

**Table 1:** Ecological data

Population	Habitat type	Vegetative cover	*H. formosa* adult density	Species richness	Predation risk
CP	Pond	27.75	8.10	7.60	36.08
GB	Intertidal	43.18	4.87	6.61	22.89
LI	Pond	24.58	1.85	6.54	33.58
ML	Pond	71.51	4.78	7.82	17.31
MS	Spring	37.72	5.39	6.21	9.00
SS	Spring	22.06	1.20	2.87	1.15
TP	Pond	34.19	1.93	4.21	41.45
TR	Intertidal	35.00	6.48	7.58	24.67
WR	Spring	53.03	48.72	7.61	4.07

Pond and spring populations also vary, on average, in the biotic environments they encounter (Table 1) (Leips and Travis 1999; Richardson et al. 2006; MacRae and Travis 2014). On average marshes have slightly higher levels of species richness than the other habitats because of their higher diversity of fishes. Lentic ponds and lotic springs have similar levels of species richness; lentic ponds have more amphibians, whereas lotic springs have more molluscs. Pond populations of *H. formosa* are characterized by lower conspecific densities and higher densities of more voracious predators. More specifically, ponds have higher densities of warmouth sunfish (*Lepomis gulosus*) and odonate naiads, especially the aeshnid *Anax junius,* whereas lotic springs have higher densities of pirate perch (*Aphrododerus sayanus*) and spotted sunfish (*Lepomis punctatus*). Warmouth exhibit predation rates 5–6 times higher than any of the other taxa, which creates a much higher predation risk for *H. formosa* in lentic ponds where the abundance of warmouth is high. There are differences among habitats in the species of submerged vegetation in which *H. formosa* seek cover, with *Myriophyllum* spp. dominating lentic ponds and the invasive *Hydrilla verticillata* dominating lotic springs. Habitats do not differ, on average, in the density of vegetation cover despite differences in the predominant species in the littoral zone. Experiments have shown that higher densities of vegetation cover make all predators less effective but that there are no differences among these species in their value as refuges when the density of vegetation is equal (Richardson et al. 2006).

We collected male *H. formosa* from each of the nine field sites in the autumn of 2009 and 2010 and during the spring of 2010. In total, we collected 209 fish from nine populations with an average sample size of 23 fish per population. Each fish was collected using dip nets then sacrificed in MS‐222 and preserved on site in a 10% formalin solution.

For this study, we collected and analyzed the body shape of male *H. formosa*. Female body shape is heavily influenced by pregnancy and by the number of broods being carried. Females carrying one or no broods appear streamlined and thin, while females carrying multiple broods have a distended abdomen and females with especially large broods can have a grossly distended abdomen. Moreover, local populations vary in body size and in their levels of matrotrophy and superfetation, both of which influence the shape of adult females, and this variation makes it easy to confound shape variation with life history variation (Soucy and Travis 2003).

### Ecological sampling

Our ecological data characterizing the different populations are derived from semi‐annual censuses of *H. formosa* populations that began May 2000 and continued through September 2010 (described in Leips and Travis 1999; Gunzburger and Travis 2004; Richardson et al. 2006; Schrader and Travis 2012; MacRae and Travis 2014). The timing of the biannual census marks the beginning (late spring) and end (early autumn) of the *H. formosa* breeding season. In these censuses, we used data from repeated sampling with a 0.5 m^2^ aluminum throw trap to estimate *H. formosa* density and incidence of all other aquatic species (including predators) at each site sampled, identified in the field to the lowest level possible (see MacRae and Travis 2014 for a complete list). With each trap sample, we visually estimated the density of vegetative cover as a percentage of the covered area. At each sampling event, we collected data from three replicate samples; our methods produce repeatable results and fail to detect only the rarer taxa in a location (Gunzburger and Travis 2004). At each sampling event, we also collected 15–20 female *H. formosa* for estimating life history parameters, including female standard length (Schrader and Travis 2012). The eleven‐year census had a sample size of roughly 66 (*n* = 66) trap censuses at each of the nine locations.

### Predation pressure

Locations in which *H. formosa* are found differ in the relative abundance of different predators that, in turn, differ in their attack rates (Richardson et al. 2006; MacRae and Travis 2014). The relative predation risk faced by these different populations cannot be described by a simple index of whether predators are present or absent or in high or low total densities. To address this challenge, we used an index of relative predation risk developed in previous studies (Gunzburger and Travis 2004; Richardson et al. 2006; Van Buskirk 2009; MacRae and Travis 2014). To arrive at this index, we first multiplied the incidence of each predator species each location on each visit (a number between 0 and 3, based on the number of trap throws in which that species was captured) by a measure of that species' predatory capacity. The predatory capacity of each species is the number of *H. formosa* an individual predator can capture and consume in 48 h in a standardized predation trial. We then add these products across the taxa detected at that site in that visit to obtain a measure of relative predation risk.

The primary predators fell into two categories, active foragers; *Lepomis punctatus* (Spotted Sunfish) and *Lepomis gulosus* (Warmouth) and ambush predators; *Aphredoderos sayanus* (Pirate Perch), and aeshnid and libellulid dragonfly larvae. Predatory capacity is derived from data presented in Richardson et al. (2006) for aeshnids (3.62), *A. sayanus* (0.96), *L. gulosus* (10.0), and *L. punctatus* (3.60). We also assigned the values estimated for aeshnids to libellulid dragonflies, which are also capable of capturing and eating *H. formosa,* particularly males (Richardson et al. 2006). Higher levels of this index are associated with lower adult densities whether on a scale of individual sampling events or averages across populations, although predation risk alone does not explain more than about 8% of the variance in density among sampling events (MacRae and Travis 2014).

### Geometric morphometrics: Body shape phenotype analysis

In order to quantify the body shape of male *H. formosa,* we made digital images of each fish using a stereoscope (Nikon SMZ100, Tokyo, Japan) with a mounted digital camera (Canon Powershot A620, Tokyo, Japan). We then employed a series of landmark‐based geometric morphometric techniques. Such geometric morphometric methods allow for the complete retention of shape data among a series of landmarks for the quantification of shape excluding the confounding effects of both size and orientation (Slice 2007). We used a two‐dimensional landmark‐based approach similar to Langerhans and DeWitt (2004). Ten landmarks were used on each fish (Fig. 1A). Images were then digitized using the software packages tpsUtil and tpsDig2 (Rohlf 2010a,b).

We used the R package GEOMORPH to perform a generalized Procrustes analysis on the landmark data to quantity multivariate shape variation (Adams and Otarola‐Castillo 2013). Using the software package, tpsRelw (Rohlf 2010c), we performed a relative warps analysis (RWA) on the digitized landmark data. Each of the RW axes from the analysis accounted for unique aspects of the overall shape variation in the dataset. We calculated the RW scores for each fish from each RW axis and used those scores as our shape variables. We also used tpsRelw to calculate the centroid size for each individual fish. Centroid size is a metric used to quantify the overall size of each fish. We retained this metric in order to estimate allometry between size and shape in statistical analysis.

### Common garden experiment

To assess the extent to which phenotypic differences among populations observed in nature may be based on genetic distinctions, we performed a common garden experiment using fish collected from ML, TP, and WR. We collected 10 pregnant females from each of the three populations in June 2009. Prior work (Schrader et al. 2011) has shown that females carry multiple sired broods in all populations, so the offspring of 10 females can harbor considerable genetic diversity. The wild‐caught adult females were kept in 5 gal (US) aquaria at a density of roughly five fish per aquarium. As females gave birth to live young, we collected all offspring from each of the adult stocked aquaria and relocated them into 5‐gal aquaria at a maximum density of 10 juvenile fish. Female *H. formosa* provision developing embryos via a placenta thereby the possibly exists for maternal effects. Each 5‐gal aquarium was provided with 100 mg of food (ground Tetramin) daily.

In the juvenile tanks, we monitored the growth and development of each individual fish until maturity. Maturity in male *H. formosa* is characterized by an elongation of the anal fin into the male intromittent organ, the gonopodium. Female *H. formosa* develop a black spot on the anal fin upon maturity. Once maturity was observed, we removed the mature individual from the 5‐gal tanks and relocated it into smaller 2.5‐gal aquaria with a total of one male and one female. All F1 males were kept in these conditions for roughly 90 days postmaturity at which point they were sacrificed and preserved in 10% formalin. All 2.5‐gal aquaria were provided 25 mg of food on a daily basis.

We raised 10 F1 males, each from a different mother, for each of the laboratory populations. We created a series of images of the F1 fish using the same protocols as the field study. We combined the images of the F1 fish with the images of the wild caught to create a dataset including both laboratory‐reared wild‐caught fish. To assess the plasticity of the body shape trait, we performed a RWA on the combined dataset that included the common garden F1 fish and their wild‐collected counterparts.

### Statistics

Our first step in analyzing the field data was to determine whether there was significant variation among populations in male shape that was not attributable to proximity. Although neutral genes do not exhibit isolation by distance within north Florida (Baer 1998b), we tested the null hypothesis that population variation in shape is related to geographic proximity. An effect of proximity could emerge if nearby pairs of populations are more likely to share an ecological or genetic history than populations separated by greater distances. To do this, we performed a generalized Procrustes analysis (GPA) to test for significant overall population variation using the GEOMORPH package for R (Adams and Otarola‐Castillo 2013). We then performed a Mantel analysis to assess whether pairwise Euclidean distances between populations in RWs 1–4 were correlated with their pairwise geographic distances in two dimensions. We used the VEGAN package (Oksanen et al. 2015) for R (R Development Core Team 2014) with 999 permutations of the distance matrices.

We then tested the effects of habitat (lotic spring, lentic pond, or tidal marsh), population identity, season (autumn or spring), and their interactions on the individual components of size and shape by performing mixed model analyses of variance on the centroid values and on the loadings of each fish on RWs 1–4 (which accounted for 80% of the total variance in shape) of the field study. We considered season and habitat as fixed effects and population identity as a random effect nested within the habitat effect. The statistical significance of each factor in these models was tested using the appropriate error terms identified from expected mean squares (Quinn and Keough 2002). The error term for habitat is the mean square for population nested within habitat; the error term for season and the interaction of season and habitat is the interaction of season and population nested within habitat. The error term for population nested within habitat and the interaction of season with population within habitat is the residual (error) mean square. We report the relative strength of significant fixed effects by their values of the index *η* ^2^, which is the ratio of the sum of squares for that factor to its appropriate error sum of squares. We report the relative strength of a significant random effect by its proportional contribution to the total variance due to random effects (i.e., $\sigma^{2}/\sigma_{T}^{2}$). We estimated variance components via restricted estimated maximum likelihood (REML).

For those shape variables for which there was significant variation at the population level, we assessed whether that variation could be predicted by habitat associations, continuous ecological variables, or both. The association of density and predation risk with the distinctions between springs and ponds make it impossible to ascribe precise values of relative importance to these factors. It is, however, possible to determine whether individual factors can predict aspects of shape variation among populations before and after accounting for the influence of other factors.

To do this, we examined the data in several ways. First, we assessed whether four continuous ecological variables that vary among populations, although not necessarily among habitats (MacRae and Travis 2014), species richness, vegetation cover, predation risk, and density, could predict shape variation independently of any association they may have with habitat. We began by assessing whether they could predict the observed average values of the centroid and each RW in the populations. This analysis revealed whether those factors could predict shape variation without taking the effects of habitat into account. We then repeated the same exercise in prediction but, as the dependent variable, we used the least squares mean value (LS mean) for each population as estimated from the best mixed model analysis of variance, as described above. This exercise revealed whether those factors could predict shape variation *after* accounting for the effect of habitat and whatever association each continuous predictor has with habitat. This process is analogous to comparing a Type I estimated effect for the continuous predictors against a Type III estimated effect. This procedure allows us to assess whether these predictors matter in their own right, independent of any confounding with habitat, or whether whatever effect they exert cannot be separated from the general effect of habitat on shape variation. In these data, predation risk and density are confounded with habitat, not species richness or vegetation cover, so this procedure is allowing us to assess the minimal and maximal roles of density and predation risk as independent predictors. Without factorial laboratory experiments (e.g., Hale and Travis 2015), there is no way to distinguish the individual effects of the several abiotic factors that vary among habitats.

Second, we assessed whether the extraordinarily high density in one population, WR, exerted excessive influence on the two sets of predictions. To do this, we repeated the analyses of species richness, vegetation cover, predation risk, and density without WR. The omission of WR eliminated confounding of average density with habitat in these data, leaving only average predation risk confounded with habitat.

For RW 1, which described the orientation of the gonopodium between a more posterior and a more anterior position (see Results), we used an additional predictor, the average body length of females in each population. We included this variable because it is implicated as a driver of sexual selection. In this species, there is sperm competition and competition among males for mating opportunities (Soucy and Travis 2003; Schrader et al. 2011). Mating in *H. formosa* occurs via forced insemination, without any attempt to elicit female cooperation; there is a very low rate of successful mating per mating attempt and a longer gonopodium with a more anterior orientation is expected to enhance the rate of success (Evans et al. 2011). In *H. formosa,* unlike most other poeciliid species tested, males prefer smaller females in dichotomous choice tests, although the reasons for this preference is unclear (Ala‐Honkola et al. 2010). These considerations led us to expect an association between a more anterior gonopodium and smaller female body size.

We assessed the predictive power of our continuous variables via all subsets regression, using the adjusted Akaike information criterion (AIC) to select the best model from among the regressions that provided significant prediction. Our goal was to determine whether one or more continuous variables could predict the average values of the centroid or a RW in these populations independently of any associations with habitat, especially in the presence of other predictors. This dataset was too small (nine populations, four predictors) for ascribing precise values of relative importance to each predictor or to discount completely a role for a predictor that is not included in the best model. However, the duration of the ecological study of these populations allowed the integration of long‐term temporal variation into the estimated average values of the predictors and offered a different perspective than what might have been possible with a short‐term survey of more populations, especially for an evolutionary inference.

In most cases, the best model had a distinctly lower value of AIC than the second‐best model (∆AIC > 4) and did not include predictors with individual *P*‐values greater than 0.05. In these cases, we report the model, the partial regression coefficients (i.e., *β*), and the proportion of among‐population variance in the average shape values accounted for by the prediction equation, adjusted for the number of parameters (i.e., $R_{\mathrm{ADJ}}^{2}$). We also report the second‐best models in those cases in which ∆AIC < 4 or in which one predictor in the best model did not have a *P*‐value less than 0.05. In this dataset, there was substantial collinearity between species richness and vegetation cover (*r* = 0.53), between predation risk and density (*r* = −0.42), and between species richness and density (*r* = 0.39) so models that include one member of the pair but not the other must be interpreted carefully. The correlation in these data between predation risk and density was driven by WR; when that population was removed, the correlation among the remaining population averages was small (*r* = 0.14).

To understand the sources of variation in the individual components of shape variation, we performed a factorial analysis of variance on the first four RW axes and centroid values from the common garden RWA. This analysis was restricted to the three populations for which we had data from both laboratory‐reared and field‐caught males. We tested for fixed effects of population, environment (field‐collected vs. laboratory‐raised), and their interaction to examine which aspects of shape might be induced by environmental effects and which aspects are maintained in a common environment and thus likely to represent local adaptation.

## Results

### Field study

**Figure 3:**
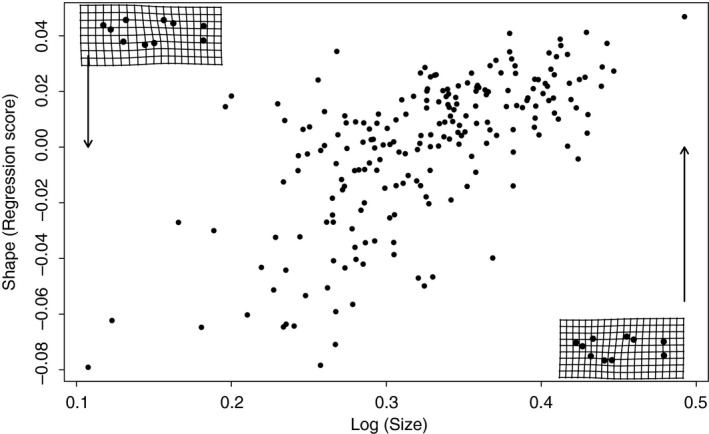
Overall *Heterandria formosa* body shape variable (from the GPA) vs. log centroid size with warped spline grids to show the shape variation. Upper left‐hand spline represents the shape of smaller fish with posteriorly positioned gonopodia; lower right‐hand spline represents the shape of larger fish with more anteriorly positioned gonopodia.

The GPA revealed significant shape variation among these nine populations (*F* = 3.105, *Z* = 1.511, *P* = 0.01) (Fig. 3). However, this variation was not significantly related to proximity (Mantel correlation = 0.24, *P* = 0.15). The distribution of distances included closely adjacent pairs of populations as well as pairs separated by considerable distances (Fig. 2), so the lack of significance is not attributable to insufficient variation in distance.

**Figure 4:**
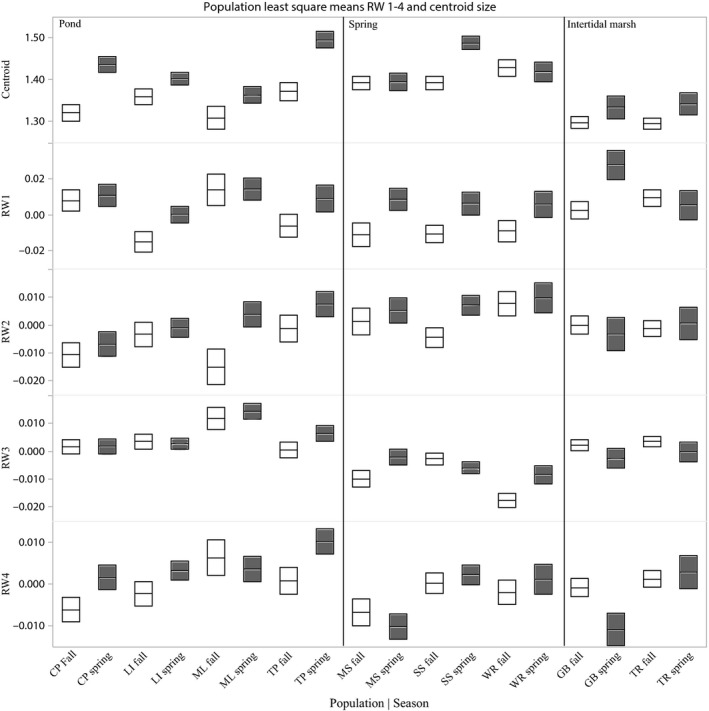
Population least square means and standard error for centroid size and RW 1‐4. Gray box indicated spring season, white boxes indicate autumn season. LS means of RW 1 are adjusted for centroid size.

Male size, as measured by the centroid size, calculated from the relative warps analysis, varied substantially between habitats, seasons, and among populations (Fig. 4). Males collected in the spring season were, on average, 5% larger than males collected in the autumn. Males from the population where they were largest (WR) were, on average, 9% larger than males from the population where they were smallest (GB and TR). Habitat (pond, spring, or tidal marsh) also had a significant effect on male size. On average, males collected from the lotic springs (MS, SS, WR) were the largest, males collected from lentic ponds (CP, ML, and TP) were intermediate in size, and males collected from tidal marshes (GB and TR) were the smallest.

Statistical analyses confirmed that season, habitat, and population affected male size (Table 2). There were strong main effects of season and habitat, with the habitat effect being stronger than the seasonal effect (*η* ^2^ = 2.35 for habitat and *η* ^2^ = 1.66 for season). There was also significant heterogeneity among populations from the same habitat, although this effect was not strong (Fig. 4; $\sigma^{2}/\sigma_{T}^{2}$ = 0.07). While the seasonal effect appeared more pronounced in ponds, the interaction of season and habitat was not significant. However, within a habitat, the seasonal effect was larger in some populations (e.g., Shepherds Spring) than in others (e.g., Wacissa River), and this generated a significant interaction between season and population ($\sigma^{2}/\sigma_{T}^{2}$ = 0.14).

**Table 2:** ANOVA table of the best models for centroid and RW 1‐4

Variable	Model	df	*F*‐ratio	*P*‐value
Centroid	Season	1	10.25	<0.025
	Habitat	2	7.06	<0.050
	Population within habitat	6	4.19	<0.001
	Season * Population within Habitat	6	2.83	<0.025
	Error	191		
RW1	Centroid	1	81.76	<0.001
	Season	1	18.30	<0.001
	Population within Habitat	6	2.88	<0.005
	Season * Centroid	1	16.63	<0.001
	Error	197		
RW2	Season	1	4.06	<0.050
	Error	207		
RW3	Habitat	2	8.57	<0.025
	Population within Habitat	6	4.54	<0.001
	Error	200		
RW4	Population within Habitat	6	4.64	<0.001
	Error	200		

**Figure 5:**
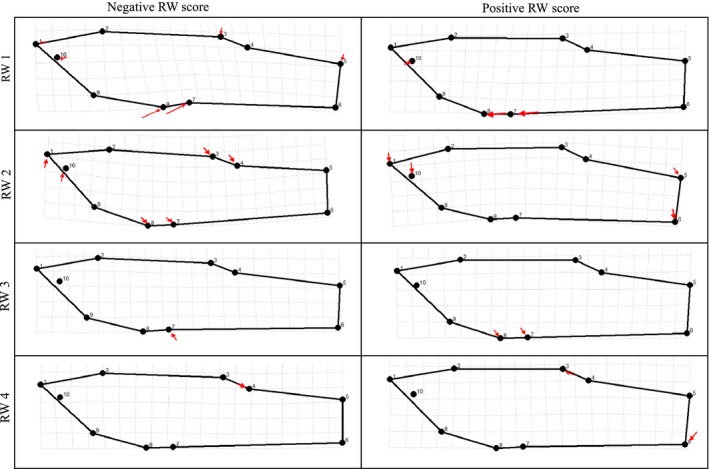
Shape change along first four axes of shape variation in the field collection data. The left column represents the extreme negative values of each of the first four RW axes. The right column represents the extreme positive values of each of these axes. The arrows indicate the direction and relative displacement (based on arrow length) of each landmark compared to the mean landmark position at the origin of each axis. The primary variation within RW 1 (from negative to positive) is a change in position of landmarks 7 and 8 which corresponds to gonopodium position. Negative RW 1 scores were associated with posterior gonopodia while more positive scores were associated with more anterior gonopodia. The negative values of RW 2 have a more upward directed snout (landmarks 1, 2, and 9) and upward point caudal fin (landmarks 4, 5, 6, and 7) when compared to the positive values. RW 3 represents a shift from a long thin caudal peduncle in the negative values (landmarks 4, 5, 6, and 7) to a shorter deeper caudal peduncle in the positive values. Variation in RW 4 is in the position of gonopodium, dorsal fin, and caudal fin.

The RWA of field‐collected males produced 16 relative warp axes with the first four axes accounting for about 80% of all shape variation. The primary shape variation accounted for by RW 1 (50% of the total shape variation) was a gradient in the origin of the gonopodium (the anal fin structures) from a more posterior to a more anterior location (Fig. 5). The variation in shape captured within RW 2 (15% of the total shape variance) described a gradient from a more dorsal orientation of the snout and caudal fin, as well as a more posterior origin to the dorsal fin, to a shape in which the snout and the caudal fin were more oriented toward the ventrum and the origin of the dorsal fin was further toward the anterior. The third relative warp (8% of the total shape variance) described a gradient in shape from fish with, longer and shallower caudal peduncles to fish with shorter, deeper caudal peduncles. The fourth RW (7% of total shape variance) described a gradient from a more posterior to a most anterior position of the dorsal fin.

Males collected from the different habitats, seasons, and populations displayed considerable variation in shape, but the pattern of this variation was different for each RW axis (Fig. 4, Table 2). Variation among populations in RW 1 was associated with variation in centroid size, so centroid size was included as a covariate in the analyses of RW 1(*η* ^2^ = 0.41). The pattern of shape variation within RW 1, adjusted for centroid size, showed that fish collected in the spring season had a more anterior position for their gonopodia than did fish collected in autumn (Fig. 4). This effect was much weaker than the association of RW1 with the centroid (*η* ^2^ = 0.09). There was also significant shape variation among populations that was maintained in both seasons, although this effect was not particularly strong ($\sigma^{2}/\sigma_{T}^{2}$ = 0.08). There was no consistent, significant association of population variation in RW 1 with habitat. The interaction of centroid and season was significant, but the magnitude of this effect was small (*η* ^2^ = 0.08).

The remaining axes of shape variation were not correlated with variation in centroid size (Table 2). There was a significant, but very weak effect of season on average score along RW 2 (Fig. 4; *η* ^2^ = 0.03): fish collected in the spring season had more dorsal orientations to their snouts and caudal fins than fish collected in the autumn. Neither habitat nor population within habitat were significantly associated with variation in RW 2. The variation in RW 3 had two significant sources of variation, habitat, and population nested within habitat. Fish collected from lotic springs had, on average, longer and thinner caudal peduncles, whereas fish collected from lentic ponds had shorter, deeper caudal peduncles with fish collected from the marshes displaying intermediate values along this gradient of shape (Fig. 4). This was a strong effect, in fact, the strongest fixed effect from all of our analyses (*η* ^2^ = 2.63). In addition to the habitat differences, there was significant and substantial variation in average RW 3 values among populations from the same habitat ($\sigma^{2}/\sigma_{T}^{2}$ = 0.20). The only significant variation detected in RW 4 was associated with population heterogeneity within each individual habitat ($\sigma^{2}/\sigma_{T}^{2}$ = 0.10).

Population identity, independent of habitat affiliation, was a significant predictor of centroid size and the components of body shape captured by RW 1, RW 3, and RW 4, so we examined whether our ecological variables (or female body size for RW 1) could predict the variation among populations in the average values of these traits.

For centroid size, the data indicated that males were smaller where species richness was greater and where densities were higher. The best model for the observed population averages ($R_{\mathrm{ADJ}}^{2}$ = 0.53) showed that males were smaller in locations with higher species richness (*β *= −0.024, *P* = 0.02) and larger where vegetation cover was higher (*β *= 0.002, *P* = 0.07). The second‐best model (∆AIC = 2) was not a significant regression and included only species richness (*β *= −0.018, P = 0.08). The best model for the LS means ($R_{\mathrm{ADJ}}^{2}$ = 0.63) showed centroid size decreasing with increasing species richness (*β *= −0.024, *P* < 0.01) and increasing with increasing density (*β *= 0.002, *P* = 0.04). When WR was removed from the analysis, the effects of vegetation cover on the observed averages and density on the LS means disappeared from the best models, leaving only species richness as a significant predictor (*β *= −0.023, $R_{\mathrm{ADJ}}^{2}$ = 0.55 and *β *= −0.022, $R_{\mathrm{ADJ}}^{2}$ = 0.63 for overall averages and LS means, respectively, both *P* = 0.01). Thus, there is no general effect of variation in cover or density on population variation in the centroid, but there is a robust effect of species richness.

**Figure 6:**
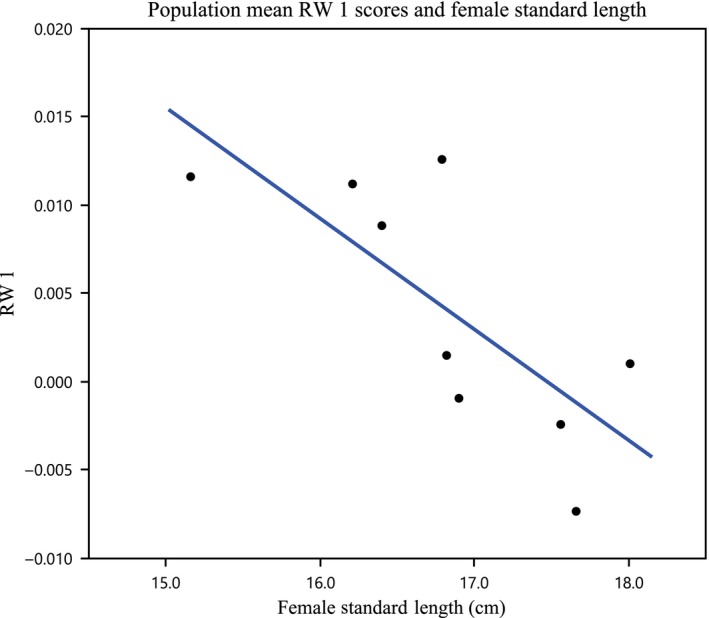
Adjusted for centroid size population least square means of RW 1 plotted against the mean standard length of females found at each population. *R* ^2^ = 0.53.

The results for RW 1 differ most between models for the observed averages and the LS means, not surprisingly, given the number of factors in the best mixed model for RW 1 and the need to adjust for the covariance of RW 1 and the centroid. The best model for the observed average values of RW 1 ($R_{\mathrm{ADJ}}^{2}$ = 0.53) indicated that the more anterior position of the gonopodium was predicted by larger centroid size (*β *= 0.139, *P* < 0.005) and higher levels of vegetative cover (*β *= 0.001, *P* = 0.03). However, a model using only centroid size (*β *= 0.108, *P* < 0.05) was almost as effective, as judged via AIC (∆AIC = 0.63), although it explained less of the variation in RW 1 ($R_{\mathrm{ADJ}}^{2}$ = 0.42). The only model that significantly predicted the variation in LS means followed the prediction that the more anterior position was predicted by smaller female body sizes (*β *= −0.006, *P* = 0.025, $R_{\mathrm{ADJ}}^{2}$ = 0.53; Fig. 6). When WR was removed, the best models in each case were virtually the same as those diagnosed when WR was included. Thus, once the relationships among RW 1, centroid, and season are taken into account, there is a robust relationship in which smaller average female body sizes predict a more anterior orientation to the gonopodium.

The population variation in RW 3 was predicted by variation in predator pressure because the robust, significant predictors were predation risk and vegetation cover, which in itself modifies the effect of predators (Richardson et al. 2006). The best model for the observed average values of RW 3 ($R_{\mathrm{ADJ}}^{2}$ = 0.83) indicated that shorter, thicker caudal peduncles (the shape toward the positive side of the axis) were predicted by decreased density (*β *= −0.00038, *P* < 0.01), increased vegetation cover (*β *= 0.00031, *P* = 0.01), and increased predation risk (*β *= 0.00022, P = 0.05). The best model for the LS means was virtually identical. When WR was removed, the effect of density disappeared. The best model for the observed averages of RW 3 without WR ($R_{\mathrm{ADJ}}^{2}$ = 0.66) included vegetation cover (*β *= 0.00029, *P* = 0.01) and predation risk (0.00021, *P* = 0.07). The next‐best model (∆AIC = 3, $R_{\mathrm{ADJ}}^{2}$ = 0.45) included only vegetation cover (*β *= 0.00027, *P* = 0.04). The best model for predicting the LS means was virtually identical to the best model for the observed averages.

No model using species richness, vegetation cover, predation risk, density, or any combination thereof was able to predict the population variation in either the observed averages or the LS means for RW 4. When WR was removed, there was no longer any significant variation among populations.

### Common garden experiment

The RWA on the common garden dataset, which included laboratory‐reared fish and their counterparts collected from the same wild populations, produced 16 RW axes of shape variation, the first four of which described gradients in shape that were similar to the gradients described by each of the first four axes in the larger set of field‐collected data. These four axes of the common garden data accounted for 83% of the total variation. Each of these axes accounted for nearly the exact same shape variation as the field study so that shape variation mirrors that seen in Figure 4. The first axis of shape variation accounted for 40% of all variation and described a shift in the position of the gonopodium from a more posterior orientation to a more anterior one (more positive RW scores). The variation captured by RW 2 (24% of the total shape variance) described a gradient from a more dorsal inclination of the snout and caudal fin to a more ventral one. Similarly, RW 3 (14% of total shape variance) described a gradient from a longer, shallower caudal peduncle to a shorter and deeper one. Finally, RW 4 captured variation in the position of the dorsal fin from a more posterior to a more anterior position (6% of total variance).

**Figure 7:**
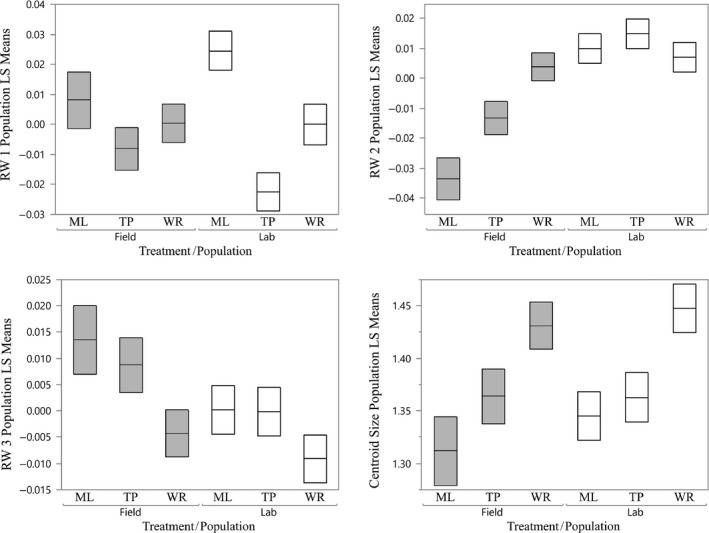
Least square means and standard error from the common garden RWA; RW 1‐3 and centroid. Gray boxes represent field fish, white represent fish from the common garden. LS means of RW 1 are adjusted for centroid size.

Population differences in male size, as measured by the centroid size in field‐collected fish, were maintained in the common environment (Fig. 7; Table 3; *η* ^2^ = 0.44). Males from WR were, on average, 5% larger than males from TP and 7% larger than males from ML whether compared in field collections or in the common garden (Table 3). There was no significant effect of rearing environment or any interaction between rearing environment and population identity.

**Table 3:** Common garden factorial ANOVA

Variable	Source	df	*F*‐ratio	*P*‐value
Centroid	Population	2	10.52	<0.000
	Environment	1	0.66	0.421
	Population * Environment	2	0.21	0.812
	Error	48		
RW1	Centroid	1	16.10	<0.001
	Population	2	9.48	<0.001
	Environment	1	0.01	0.62
	Population * Environment	2	2.27	0.11
	Error	47		
RW2	Population	2	3.56	0.036
	Environment	1	40.26	<0.001
	Population * Environment	2	4.76	0.013
	Error	48		
RW3	Population	2	4.39	0.018
	Environment	1	4.84	0.033
	Population * Environment	2	0.35	0.708
	Error	48		
RW4	Population	2	3.15	0.051
	Environment	1	1.93	0.171
	Population * Environment	2	0.93	0.401
	Error	48		

As in the field data, variation among individuals in RW 1 was associated with variation in centroid size (Fig. 7; Table 3; *η* ^2^ = 0.34) and so analyses of RW 1 were performed as analyses of covariance that took the influence of the centroid into account. As was the case for the centroid, population differences in RW 1 in field‐collected fish were maintained in the common environment (Fig. 7; Table 3; *η* ^2^ = 0.40). Fish from WR and ML had a more anteriorly placed gonopodium regardless of whether they were wild‐caught or F1 fish, and fish from TP had a more posteriorly placed gonopodium. The lack of a significant overall effect of environment on the adjusted mean values of RW 1 masks an interesting pattern in these data: average‐adjusted RW1 values were similar for F1 WR and field‐caught WR but appeared to differ in opposite directions for males from TP and ML. The interaction was not significant, and the within‐group variation is sufficiently large that any potential interaction appears weak in any case (*η* ^2^ = 0.10).

In contrast with the results for the centroid and the adjusted means of RW 1, the analysis of RW 2 revealed a significant effect of rearing environment (Fig. 7, Table 3, *η* ^2^ = 0.84). The data demonstrated that the population differences seen in field‐collected fish were not manifested in laboratory‐reared fish, indicating that population differences seen in the field were almost entirely environmentally determined. The comparatively weak, statistically significant effect of population identity (*η* ^2^ = 0.15) was based entirely on the substantial differences seen in the field‐collected fish. The contrast between these large differences and the lack of any differences in the F1 fish produced the significant interaction between rearing environment and population identity (Table 3; *η* ^2^ = 0.20).

The pattern of variation in RW3 indicated that differences seen in wild‐caught males are maintained in a common environment, even though they are much less pronounced there (Fig. 7; Table 3). This is the opposite pattern from that seen in RW1, in which differences among populations are more pronounced in laboratory‐reared males. The effect of population identity was somewhat stronger than that of rearing environment (*η* ^2^ values of 0.18 and 0.10, respectively). There was no significant interaction between population and rearing environment; F1 males from the three populations displayed the same rank order of average position along this axis as did field‐collected males.

The comparison of variation in RW4 between wild‐caught males and laboratory‐reared males produced a similar pattern to that displayed in RW1 (Fig. 7, Table 3). For RW4, there was a significant effect of population, although a weak one (*η* ^2^ = 0.13), with more pronounced variation among populations in laboratory‐reared than in wild‐caught males.

## Discussion

Associations between ecological variables and phenotypic features across populations are often viewed as suggestive evidence for local adaptation (Endler 1986; Reznick and Travis 1996). This is particularly true for the body morphology of fish, which has a direct effect on locomotion and, ultimately, fitness (Collin and Fumagalli 2011; Fu et al. 2013).Various aspects of a fish's local environment can be agents of selection on body size and shape, including abiotic factors like water flow and the physical structure of the habitat (Franssen 2011; Kane and Higham 2012) or biotic ones like evading attacks from predators (Langerhans and Makowicz 2009) and the effects of competing species on where food is available and how it can be harvested (Svanbäck and Eklöv 2002). Resolving whether associations between ecological variables and phenotypic features reflect local adaptation depends on (1) establishing whether population differences are at least in part based on genetic differences and then (2) demonstrating how the individual ecological variables combine to select for specific phenotypic values.

We address both requirements in this study. Our RWA allowed us to dissect the variation in body shape, and these results suggest that population‐level variation in male size (centroid) and the orientation of the gonopodium (RW 1) has a substantial genetic basis and the population‐level variation in the shape of the caudal peduncle (RW 3) and the orientation of the dorsal fin (RW 4) has a significant though weaker genetic basis. Our results from both the field and common garden experiment suggest that the orientation of the snout and tail toward dorsum or ventrum (RW 2) is determined largely by environmental effects on the phenotype.

Given that we used laboratory‐born offspring of wild‐caught females, it is possible that the persistence of size and shape differences among males from different populations raised in a common laboratory environment is entirely due to environmentally induced maternal effects on ontogeny and not to genetic differences. Density‐induced maternal effects on offspring size at parturition and offspring growth rate have been described in two of these populations (Leips et al. 2009, 2013). If differences in growth rate generate shape differences via the developmental connections among features and overall growth, then density‐induced maternal effects on growth rate could masquerade as genetic effects on shape in a study like ours. However, if density‐induced maternal effects were the sole source of shape differences, we would expect ML to be intermediate between TP and WR in the various components of size and shape, given that its long‐term average density is between those two. This was not the case for the centroid and RW 1, which are the components with the largest population effect. This does not preclude the possibility that environmentally induced maternal effects of an unknown nature are masquerading as genetic effects in these data.

Trait variation among populations was not distributed randomly with respect to ecological factors. We found that males are larger in lotic springs (centroid) and, in addition, smaller in locations with greater species richness. Males have a more anterior orientation of the gonopodium where females are smaller (RW 1). Fish from lotic springs, where predation pressure is lowest, had longer, thinner caudal peduncles (RW 3). In parallel fashion, looking among populations within habitats, fish had longer and thinner caudal peduncles in locations with lower predation pressure. We found no associations of any kind for predicting population variation in the position of the dorsal fin (RW 4).

Several lines of evidence suggest that the variation in male body size may be adaptive. First, our results on male size are consistent with prior studies of this species. Variation in the size of field‐collected males was also reported in Schrader et al. (2012); in those data as well as ours (Fig. 4), males from lotic springs were typically larger than males from lentic ponds. Our common garden results match those from other studies of stocks from WR and TP in a common environment, which found that males from WR matured at a larger size than males from TP (Leips et al. 2000; Hale and Travis 2015). Second, the genetic distinctions among populations in male size are likely greater than these common garden results suggest. Males grow more slowly and are smaller at higher densities and the densities at lotic springs are, on average, higher than those at lentic ponds (Leips and Travis 1999; Soucy and Travis 2003; Richardson et al. 2006; MacRae and Travis 2014). As a result, the observed range of male size variation among habitats is likely an underestimate of the real range of genetic distinctions for body size among males in different habitats. Third, the pattern of body size variation among populations matches the direction of divergent selection indicated by studies of ecological interactions. Warmouth sunfish predation (*Lepomis gulosus*), which is a predominant force of mortality in lentic ponds, selects against larger males (Richardson et al. 2006), whereas competition, which is more intense in the lotic springs with higher densities and lower predation risks, selects for larger body sizes (Leips et al. 2013).

Population differences in the orientation of the gonopodium (RW 1) were significantly associated with female standard length. The existing evidence on the mating system and the preference of males for smaller females indicates that this result likely reflects the results of intrasexual selection. A male *H. formosa* uses his gonopodium by rotating it anteriorly so that it points directly ahead (Fig. 1B). Males typically swim up from beneath a female to attempt to mate without first eliciting female cooperation. In other species of poeciliid, individual fish with longer gonopodia have been shown to increase the rate in which males make contact with the female reproductive duct and this advantage of a longer gonopodium is especially striking in species, like *H. formosa,* in which males rely primarily on forced insemination (Evans et al. 2011; Heinen‐Kay and Langerhans 2013). One benefit of a longer gonopodium is that when it is extended to or past the eye, such as in some male *H. formosa,* it may be advantageous by providing a visual cue for males to work with when attempting to mate (Greven 2005). An anterior shift in the position of the gonopodium places it in a position closer to the eye, thereby making the entire structure functionally larger, and possibly increasing the effectiveness of forced mating. In this way, an anterior shift in gonopodium position may reflect an adaptation to higher levels of male–male competition in *H. formosa*.

Results from this study suggest that the population variation in RW 3, which described a gradient from longer, thinner caudal peduncles to shorter, deeper ones, is an adaptive response to predation pressure. First, although there was clearly an environmental effect on RW 3, it was weaker than the differences among populations, which were maintained in F1 males in a common environment. Second, the pattern of population variation in our data matches patterns seen in other studies. For example in some taxa, longer more streamlined caudal peduncles offer benefits in flowing waters, whereas shorter and deeper ones improve maneuverability and foraging economy in lentic ones, especially in thicker vegetative cover where increased habitat complexity alters flow rate and can affect the locomotion ability of fish (Franssen 2011; Ruehl et al. 2011; Gaston and Lauer 2015). Many studies have described an association between caudal peduncle shape and predation risk because of the value of a deeper tail for rapid acceleration away from a predator (Ghalambor et al. 2004; Langerhans et al. 2004; Walker et al. 2005; Hendry et al. 2006; Langerhans and Makowicz 2009). In these locations, the lentic ponds do, on average, present male *H. formosa* with a higher predation risk. The stouter peduncle is presumably selected against in lower predation habitats like lotic springs because it decreases the ability to gather resources in a high flow environment or find a mate (Ghalambor et al. 2004).

While the gradient from a dorsal orientation of snout and tail to a ventral one was the second axis of shape variation, there was no evidence that this gradient was anything but an environmentally created one. Only season of collection predicted shape variation in the overall field collection and this effect was itself quite weak. While a subset of populations (ML, TP, and WR) showed some differences among field‐collected males, these vanished in the common environment. The observable variation may reflect seasonal or other transient differences in food availability during ontogeny that channel foraging patterns that, in turn through muscular exercise and re‐shaping, create morphological differences.

One striking result from our field‐collected data was the prevalence of seasonal effects on size and shape. Season of collection influenced centroid size, RW 1, and RW 2, although the effect was strong only for the centroid (*η* ^2^ = 1.66, compared with values of 0.09 and 0.03 for the other variables). It is easy to speculate about the seasonal effect of centroid size: the phenology of *H. formosa* life histories (Travis et al. 1987; Leips and Travis 1999) indicates that males collected in May (spring season) may be older and therefore larger than males collected in September (autumn).

While we were unable to estimate the relative importance of each of the possible agents of selection for which we had data, we have shown that they are acting either in concert or in opposition on different components of the integrated phenotype, body shape. That different agents of selection may be important for different components of shape is not surprising when one considers that different agents of selection have long been known to act on different phenotype traits. Shape, however, presents some novel challenges because it can change with an animal's age, it varies between genders, and, as we have shown, different components of shape are molded by different combinations of genetic and environmental effects. Our results illustrate the importance of a detailed ecological understanding of an organism's natural history and selective milieux; the next steps in understanding these patterns are to obtain an equally deep understanding of how shape in these animals develops under the influence of different genotypes and environmental influences.

## Data accessibility

Data to be archived on Dryad Digital Repository: doi:10.5061/dryad.q5v77.

## Conflict of Interest

None declared.
